# Introducing the Dish Soap Protocol: A Unified Approach for Multi‐Modal Intracellular Staining

**DOI:** 10.1002/cpz1.70206

**Published:** 2025-09-19

**Authors:** Oliver T. Burton, James Dooley, Adrian Liston

**Affiliations:** ^1^ Department of Pathology University of Cambridge Cambridge UK; ^2^ These authors contributed equally to this work

**Keywords:** flow cytometry, GFP, immune phenotyping, intracellular staining, spectral flow cytometry

## Abstract

Recent advances in dyes and cytometers have seen an exponential increase in the ability to perform multidimensional flow cytometry. As we increase our capacity to extract information from cells, the need to acquire different types of data simultaneously from the same cells becomes limited by fixation buffer compatibility. Accessing the intracellular compartments for staining, particularly with large molecular weight structures such as antibody–fluorophore conjugates, requires a balance between solubilizing lipids to create entry points through the membranes, while using crosslinking fixatives to maintain structural integrity of the cell and prevent the loss of intracellular contents. Unfortunately, common fixation protocols result in a trade‐off, where preservation of fluorescent proteins and accessibility of nuclear staining are often diametrically opposed. In this article, we detail the impact of various fix‐perm reagents on key features, such as nuclear staining, green fluorescent protein (GFP) retention, intracellular cytokine staining, epitope retention, scatter profiles, cell recovery, and fluorophore stability. We provide a protocol for the use of “Burton's Best Buffer”, which is readily made at 100‐fold lower cost than commercial buffers, which can be used to overcome the limitations of current fixation buffers and achieve simultaneous efficient detection of transcription factors, cytokines, and endogenous fluorescent proteins, among other uses. © 2025 The Author(s). Current Protocols published by Wiley Periodicals LLC.

**Basic Protocol**: Dish soap protocol

## INTRODUCTION

Fluorescent proteins can provide easy‐to‐use, accurate readouts of cell states and have proven to be invaluable tools for both transgenic animal research and in human immunology (e.g., CAR T cell transduction monitoring). Many of the most informative markers are intracellular in origin, such as transcription factors, cytokines, and endogenous fluorophores used to mark engineered cells. Accessing the intracellular compartments for staining, particularly with large molecular weight structures such as antibody‐fluorophore conjugates, requires creating holes in the membranes while preventing the cell from completely disintegrating or losing cytoplasmic contents. This often achieved using a formaldehyde‐based fixative to crosslink proteins, creating a rigid scaffold, then solubilizing lipids with detergents such as saponin. However, detection of intranuclear markers, notably transcription factors, requires extensive permeabilization of the cell in order to allow the macromolecular antibody‐fluorophore conjugates to access the proteins in the nucleus, and can be blocked by excessive crosslinking. By contrast, when seeking to preserve cytosolic fluorophores, the conditions optimal for intranuclear staining often completely ablate detection, with loss of cytoplasmic contents due to insufficient crosslinking and high use of solubilizing factors. This has rendered the simultaneous detection of transcription factors and fluorescent protein reporters challenging. This technical problem limits our ability to answer key scientific questions.

Here we set out to screen for a fixation‐permeabilization solution that would allow for better combined detection of nuclear staining and fluorescent protein signal retention than is currently possible. Two key prior publications have addressed the combination of transcription factor staining and green fluorescent protein (GFP) retention, both focusing on Foxp3 Treg detection (Grupillo et al., 2011; Heinen et al., 2014). Grupillo et al. (2011) used 4% paraformaldehyde (PFA) prior to proceeding with both the fix/perm and permeabilization steps of the eBioscience Foxp3 kit. Heinen et al. (2014) demonstrated that while this approach preserves fluorescent protein reporters, it is largely ineffective for transcription factor staining. Our experiences are entirely consistent with the results described by Heinen et al. (2014) in this regard. However, Heinen et al. (2014), found that fixation with 2% formaldehyde followed by permeabilization with the eBioscience permeabilization buffer (no additional fix/perm step) permitted enhanced transcription factor staining, with a modest decrement in GFP retention. However, by using both direct Foxp3 expression and a Foxp3 reporter, we find that this protocol only partially enables Foxp3 detection, and that staining for other transcription factors (except for T‐bet) is greatly reduced or negligible, even with overnight staining to enhance detection. Furthermore, 2% formaldehyde crosslinks many epitopes (Hoffman et al., 2015), preventing accurate staining post‐fix for many CD antigens and even transcription factors, such as Helios. Therefore, we sought to find a better solution, leading to the optimization of “Burton's Better Buffer”, for simultaneous detection of transcription factors and fluorescent reporters. This buffer, based on cheap dishwashing detergent, is fortuitously also compatible with most intracellular staining protocols, such as intracellular cytokine staining, other than phospho‐flow.

*CAUTION*: Fixatives mentioned in this protocol contain paraformaldehyde and should be used with appropriate safety considerations in a fume hood.

*NOTE*: These buffers are not optimal for either transcription factor staining or GFP retention in isolation.

*NOTE*: All protocols involving animals must be reviewed and approved by the appropriate Animal Care and Use Committee and must follow regulations for the care and use of laboratory animals. Appropriate informed consent is necessary for obtaining and use of human study material.

## DISH SOAP PROTOCOL

This protocol involves the use of a commercial product, Fairy dish soap, to faciliate preservation and permeabilization of cells for intracellular flow cytometry assays.

**Figure 1:**
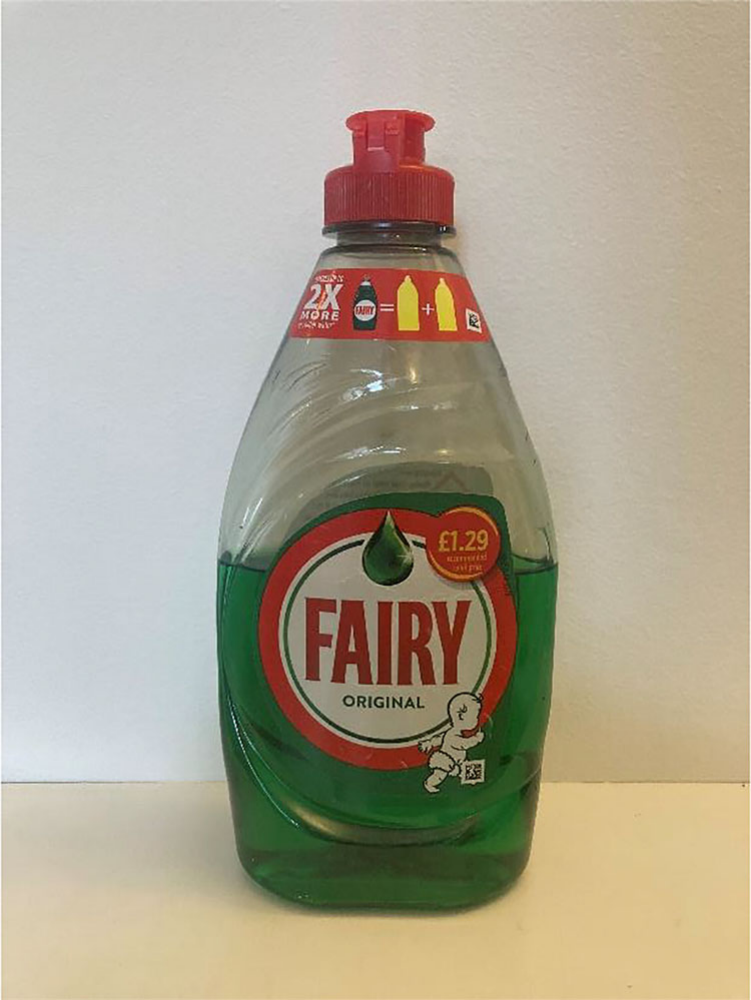
Fairy dishwashing liquid detergent.

*NOTE*: In the United Kingdom, the Proctor & Gamble dishwashing liquid detergent is marketed under the name Fairy (see Fig. 1), but in other countries it may be available as Dreft, Dawn, Yes, or JAR. We have not tested these variants. Fairy is a green, viscous liquid containing surfactants. Other washing up liquids and scientific surfactant preparations behave somewhat similarly, but do not provide the same quality of results. In the process of developing this protocol, we have used regular strength “Original” Fairy (United Kingdom), Fairy Ultra (United Kingdom), and Dreft (Belgium) with equivalent results.

### Materials

Fixative (see recipe)
Perm buffer (see recipe)
FACS buffer (see recipe)
Block, e.g., Fc receptor block or serum

Centrifuge
Fume hood
Flow cytometer

Additional cells and materials for performing surface staining

1 Perform surface staining as normal: Count cells, block, stain, wash.
2 After surface staining, centrifuge cells 5 min at 400 to 600 × *g*, room temperature. Discard the supernatant.
3 Resuspend the cell pellet in 200 µl fixative. Incubate 30 min at room temperature in the dark.
  Perform this step in a fume hood.
4 Centrifuge 5 min at 600 × *g*, room temperature. Remove supernatant.
  Dispose of formaldehyde‐containing buffers appropriately.
5 Resuspend in 100 µl perm buffer. Incubate 15 to 30 min at room temperature. Blocking may be done at this stage by adding the block to the perm buffer.
6 Wash twice in FACS buffer.
7 Stain overnight in FACS buffer at 4°C.
  Additional permeabilization is neither necessary nor recommended.
8 Wash twice in FACS buffer.
9 Acquire samples on flow cytometer.

## REAGENTS AND SOLUTIONS

### FACS buffer

1 L of 1× phosphate‐buffered saline (PBS) (Current Protocols, 2006)
25 ml fetal bovine serum (FBS) (TICO Europe, cat. no. FBS‐EU500; various sources are acceptable)
4 ml of 0.5 M UltraPure EDTA, pH 8.0 (Thermo Fisher, cat. no. 15575020)
Mix and store up to 2 weeks at 4°C
  For longer term storage, 10 ml of 10% (v/v) sodium azide may be added (0.1% final). Bovine serum albumin (BSA) may be substituted for FBS. In this case, add 5 g BSA to reach 0.5% (w/v) final.

### Fairy in PBS, 5%

Dispense 9.5 ml PBS (Current Protocols, 2006) into a 15‐ml Falcon tube
Cut the tip of a P1000 (blue) pipette tip with scissors to widen the bore
Pipette 500 µl Fairy dishwashing liquid detergent (see Fig. 1) into the 15‐ml tube
Cap and invert several times to mix
Store up to 6 months at room temperature

### Fixative (2% formaldehyde with 0.05% Fairy and 0.5% Tween)

5 ml of 4% formaldehyde
4 ml PBS (Current Protocols, 2006)
1 ml of 5% Tween‐20 (see recipe)
100 µl of 5% Fairy (see recipe)
Optional: add 200 µl of 5% Triton X‐100 (0.1% final; see recipe)
Store up to 6 months at room temperature
  Fixative and detergent solutions may be stored at room temperature. We have not observed any loss of performance.
  As Triton X‐100 has now been banned from sale in the European Union due to concerns regarding endocrine‐influencing breakdown products, we note that the Triton may be omitted with similar results. The concentrations of detergents can also be modified up or down by a factor of 2 with only modest impact on the results.

### Perm buffer (PBS with 0.05% Fairy)

9 ml PBS (Current Protocols, 2006)
100 µl of 5% Fairy (see recipe)
Store up to 6 months at room temperature
  At this low concentration, the solution should not be bubbly unless agitated.

### Triton X‐100 in PBS, 5% (optional)

Dispense 9.5 ml PBS (Current Protocols, 2006) into a 15‐ml Falcon tube
Cut the tip of a P1000 (blue) pipette tip with scissors to widen the bore
Pipette 500 µl Triton X‐100 (e.g., Sigma‐Aldrich, cat. no. T9284) into the 15‐ml tube
Cap and invert several times to mix
Store up to 6 months at room temperature

### Tween‐20 in PBS, 5%

Dispense 9.5 ml PBS (Current Protocols, 2006) into a 15‐ml Falcon tube
Cut the tip of a P1000 (blue) pipette tip with scissors to widen the bore
Pipette 500 µl Tween‐20 (e.g., Sigma‐Aldrich, cat. no. P1379) into the 15‐ml tube
Cap and invert several times to mix
Store up to 6 months at room temperature

## COMMENTARY

### Initial Optimization and Screening: GFP + Foxp3

To start, we chose to focus on retaining GFP while detecting Foxp3. For GFP, we selected a weakly expressed iCas9 – IRES – GFP construct expressed in the Rosa26 locus under the control of a CD4Cre driver (Lee et al., 2001; Platt et al., 2014). To determine the accuracy and completeness of Foxp3 staining, we use a Foxp3‐Thy1.1 cell surface reporter as “ground truth” (Liston et al., 2008). Over the last eight years, we have tested commercial kits from BD, Thermo Fisher and BioLegend as well as >1000 combinations and concentrations of fixatives and permeabilization agents. Broadly, we determined that GFP is better preserved with higher concentrations of PFA in the absence of any detergent, and that Foxp3 staining works best with lower concentrations of fixative in combination with stronger detergent. Permeabilization without prior formaldehyde‐based fixation did not preserve GFP, in contrast to published results (Jensen et al., 2010). While the general parameters required to achieve GFP preservation and nuclear staining are at odds, we find that there are inflection points where inclusion of a mild detergent in a moderately strong fixative can retain enough GFP to preserve the staining pattern while allowing complete detection of Foxp3.

**Figure 2:**
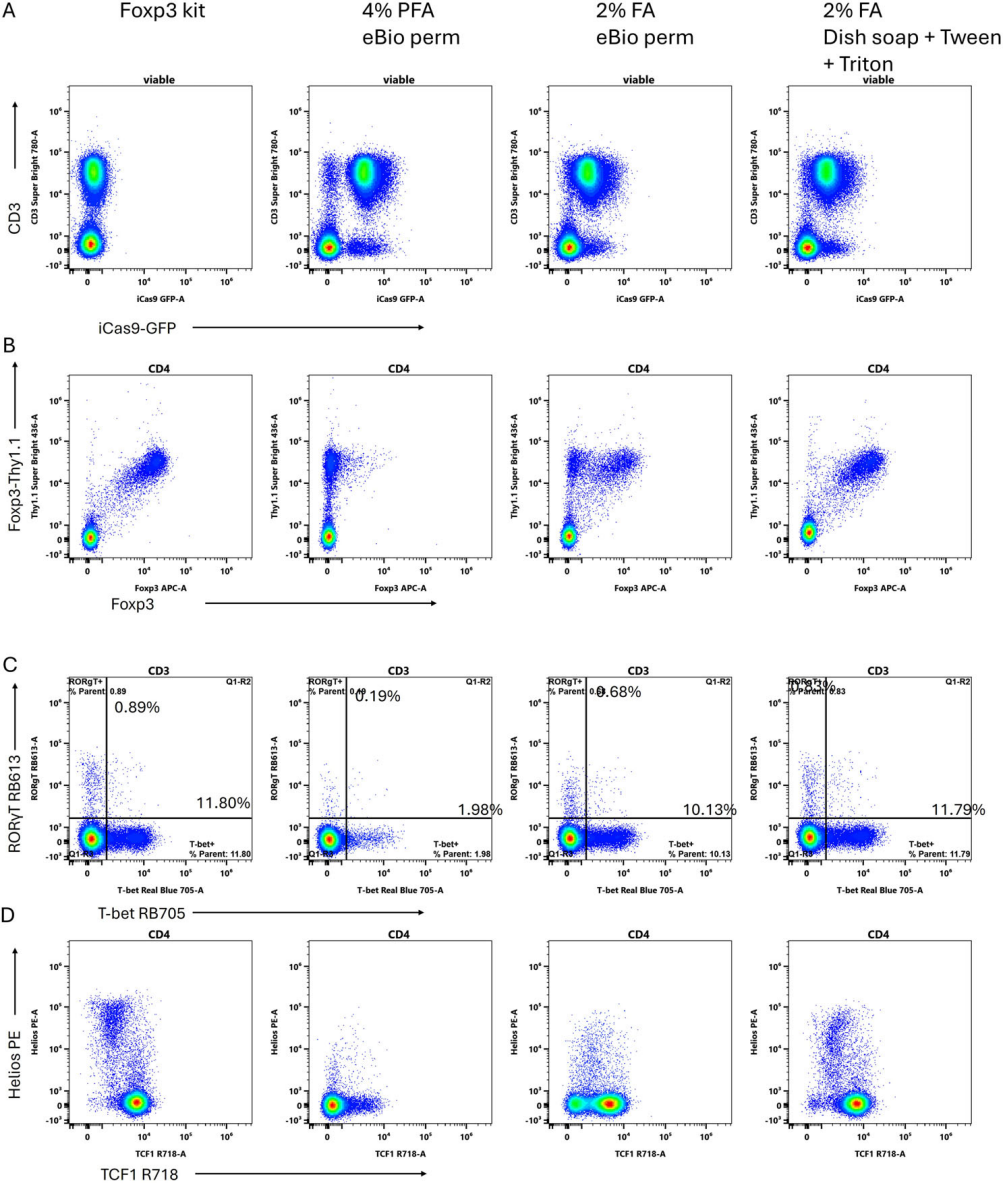
Development of a new fixative. (**A**) Comparison of GFP retention after fixation and permeabilization using the commercial Foxp3 kit, the two previously published methods and the new dish soap protocol. GFP expression in CD4Cre iCas9‐GFP mouse splenocytes is expected in nearly all CD3^+^ T cells. (**B**) Foxp3 staining in mouse CD4 T cells with overnight staining. The Thy1.1 is expressed from the Foxp3 locus and thus provides a reference for which cells should stain for Foxp3. (**C**) Nuclear staining for RORγT and T‐bet in mouse CD4 T cells using the different methods. The Foxp3 kit provides the gold standard here. (**D**) Detection of TCF1 and Helios in mouse CD3^+^ T cells. Plots are shown gated on viable single CD3^+^ cells, followed by CD4 gating where indicated.

In particular, a solution of 2% formalin admixed with 0.05% Fairy dish soap, 0.5% Tween‐20, and 0.1% Triton X‐100, was effective at allowing GFP and Foxp3 to be detected simultaneously. When this combination was used as a fixative followed by a permeabilization step with 0.05% Fairy dish soap in PBS, the GFP signal pattern was preserved (Fig. 2A), albeit with a modest reduction in signal intensity compared to cells fixed with 4% PFA or fresh, unfixed cells (not shown, equivalent to 4% PFA). With the dish soap protocol, the pattern of Foxp3 protein detection matched the Foxp3‐Thy1.1 surface reporter, as with the gold standard eBioscience Foxp3 kit (Fig. 2B). In contrast, the published 4% PFA and 2% formaldehyde protocols showed variable detection of nuclear Foxp3 protein. Other key immunological transcription factors, such as T‐bet, RORgT, TCF1, and Helios, were detected relatively well (Fig. 2C‐D). In general, the detection of transcription factors tends to be slightly worse with the dish soap protocol than with the Foxp3 kit, which is optimized for transcription factor staining. Similarly, preservation of fluorescent protein signals is impaired relative to the 4% PFA fixation. Neither of those approaches allows for the combined detection of GFP and transcription factors, though.

**Figure 3:**
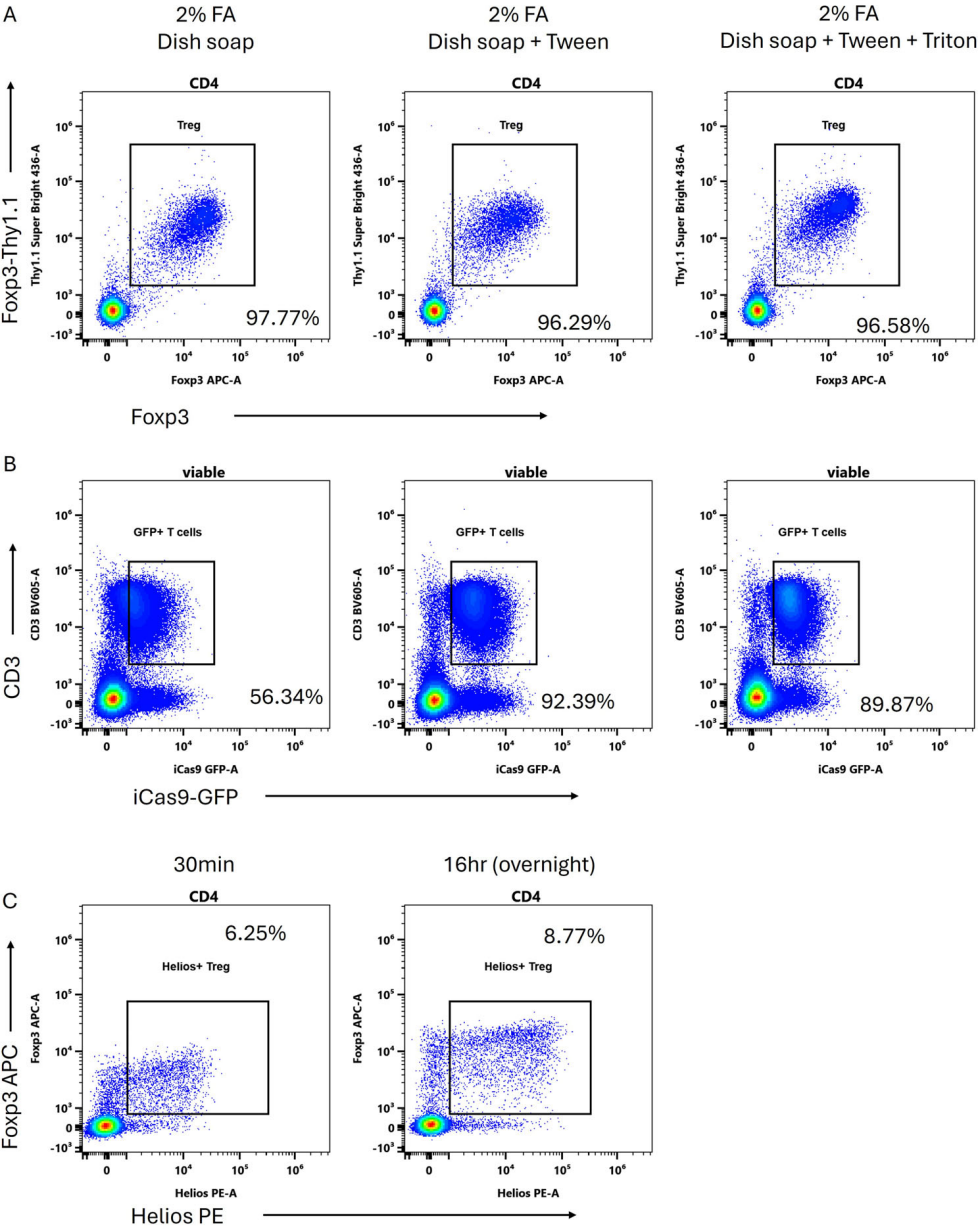
Variations on the protocol. (**A**) Impact of the addition of Tween‐20 and Triton‐X‐100 on Foxp3 detection in mouse CD4 T cells. (**B**) Impact on GFP signal preservation. (**C**) Length of staining time influences Foxp3 and Helios detection in mouse CD4 T cells using the dish soap protocol. Gated on viable CD3^+^CD4^+^ cells.

In Figure 3, we provide examples of other workable fix‐perm reagent combinations that we found, all based around 2% formaldehyde. Adding 0.05% Fairy dish soap alone allows for quite good transcription factor staining but reduces GFP preservation (Fig. 3A‐B). The addition of 0.5% Tween‐20 to this reduces GFP loss, with a modest impact on Foxp3 staining. The final combination of 0.05% Fairy, 0.5% Tween‐20, and 0.1% Triton‐X‐100 provides a minor improvement in transcription factor staining and a minor loss in GFP. Either of the latter two combinations works well. In all cases, 0.05% Fairy in PBS was used as a permeabilization incubation step after the fix‐perm step. We note that with all of these combinations, extended staining time (Whyte et al., 2022) provides marked improvements in transcription factor detection with no loss in GFP signal (Fig. 3C and not shown).

### Secondary Screening: Feature Preservation

**Figure 4:**
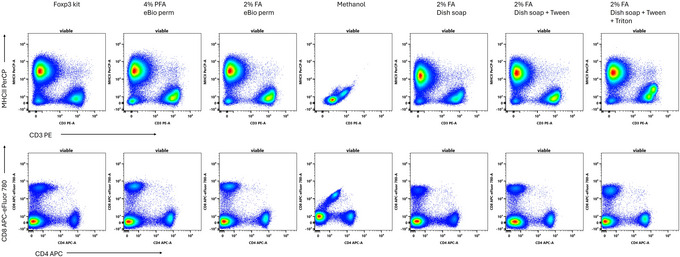
Comparison of fixative impact on fluorophore stability. Mouse splenocytes were stained with protein‐based fluorophores (PE, PerCP, APC, and APC‐eFluor780) prior to fixation and permeabilization using various methods. Samples were incubated overnight prior to acquisition. Gated on viable single cells.

In multi‐color flow cytometry, reliable discrimination of fluorophore signals determines accurate results. Several fluorophores, however, are prone to chemical alteration when subjected to fixatives, resulting in either reduced or altered fluorescence (Blanks, 2023; Krutzik et al., 2011). We tested the impact of the dish soap‐based fix‐perm reagents on different classes of fluorophores. In Figure 4 we present examples of the impact on the most problematic cases we observed. The use of Fairy dish soap without the addition of Tween‐20 resulted in a modest loss of signal for PerCP‐based conjugates (Fig. 4). Adding Tween (the recommended combination) largely corrects this. We do not observe increased tandem breakdown with the dish soap protocol, nor do we observe loss of fluorescence as seen with methanol‐based fixation (Fig. 4).

Additional key considerations for fixatives include how well epitopes are preserved for post‐fix staining, whether scatter profiles are retained to allow discrimination of cells based on size and granularity and, particularly with fix‐perm reagents, whether substantial numbers of cells are lost during the process. The Foxp3 kit minimizes epitope crosslinking, allowing post‐fix staining of many markers, but it causes a loss of essentially all scatter characteristics and typically reduces cell recovery by factors of 2‐ to 3‐fold. At the other end of the spectrum, fixation with PFA preserves cell numbers and scatter, with extensive crosslinking that prevents accurate post‐fix staining of many markers. The dish soap protocol provides recovery comparable to PFA fixation, moderate loss of epitopes due to cross‐linking and permits retention of scatter, although the cells appear a bit smaller than with PFA‐based fixation. These data are not included here but are available in the files uploaded to Mendeley Data (see Data Availability Statement).

### Cytokine Detection

**Figure 5:**
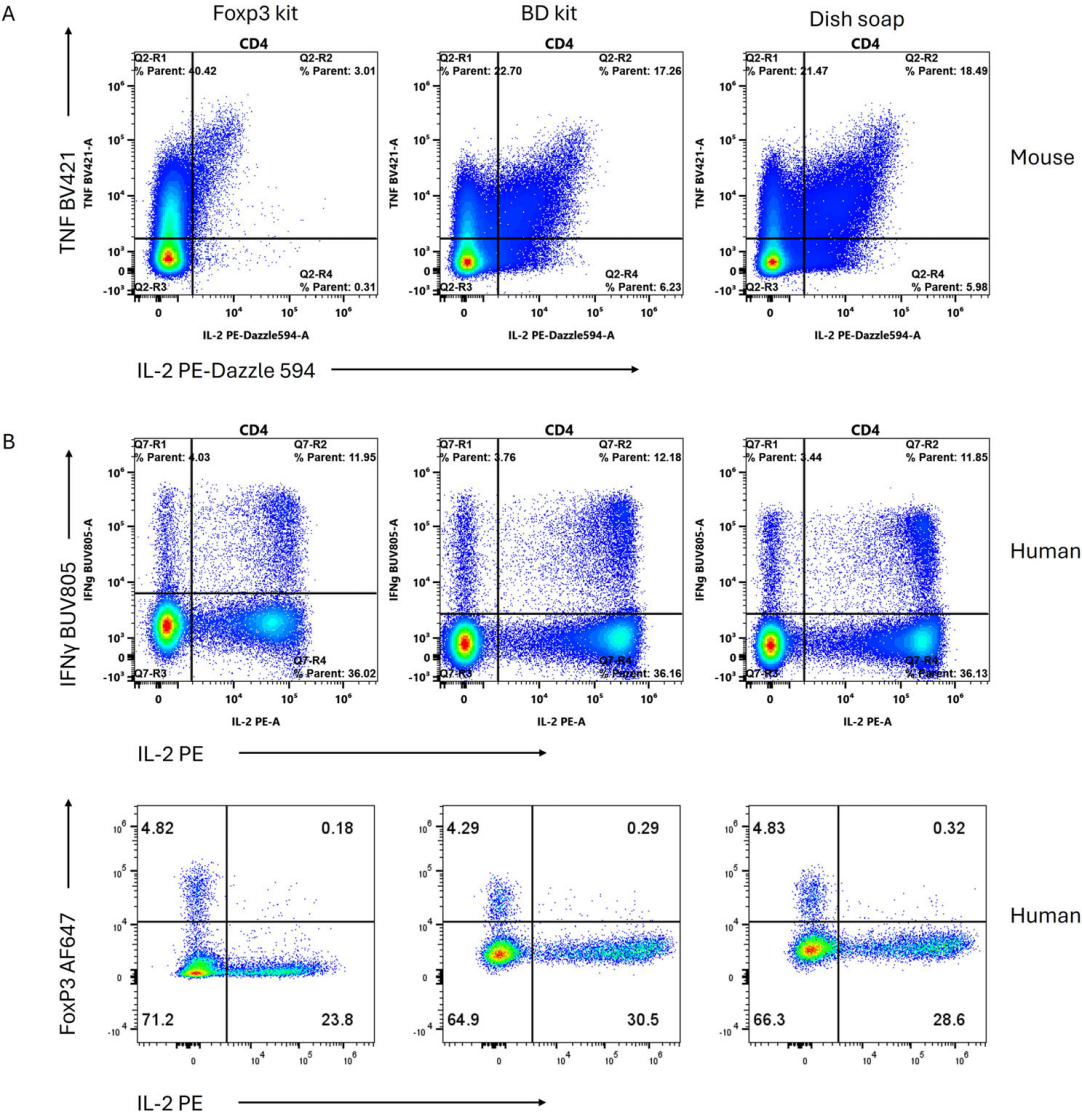
Suitability of fixatives for intracellular cytokine detection. (**A**) Staining for TNF and IL‐2 in mouse CD4 T cells after using the eBioscience Foxp3 kit, the BD Cytofix/Cytoperm kit or the dish soap protocol. (**B**) Staining for IFNg and IL‐2 or FoxP3 and IL‐2 in human CD4 T cells. Samples were stimulated for 4 hr with 100 ng/ml PMA, 750 ng/ml ionomycin, and 2 µg/ml brefeldin A prior to staining.

Intracellular cytokine staining taps into the cell‐to‐cell communication network, allowing us to eavesdrop on the conversations driving immune responses. Detecting the cytokines can be tricky since the expressing cells may be extremely infrequent, and the protocol requires the secretion to be blocked to build adequate levels for detection within the cells. There are several optimized approaches for intracellular cytokine detection, and we direct the reader to a few key publications if the only interest is in detecting cytokines (Lamoreaux et al., 2006; Lovelace & Maecker, 2018; Mair & Tosevski, 2014). In our experience, fixatives that are optimized for cytokine staining, such as BD's Cytofix‐Cytoperm and BioLegend's CytoFAST kits, permit excellent preservation of fluorescent proteins, but are not designed to allow transcription factor staining. We note that the BD Cytofix‐Cytoperm kit, when combined with overnight staining, does provide permit decent transcription factor staining for human cells (data not shown but are available on Mendeley Data). In contrast, kits designed to detect transcription factors often cause extensive permeabilization of cellular membranes, releasing cytokines and preventing them from being detected well. In Figure 5, we demonstrate the utility of the dish soap protocol for intracellular cytokine staining on both mouse and human cells. For mouse cells, IL‐2 is among the most sensitive to fixation conditions. With the eBioscience Foxp3 kit, IL‐2 is nearly entirely lost, whereas substantial levels are detected when the BD Cytofix‐Cytoperm kit or the dish soap protocol are used (Fig. 5A). Similar losses of cytokines were observed with BD's Transcription Factor and Transcription Factor Phospho buffer sets (data not shown). In our experience, IL‐2, TNF, IL‐4, and IL‐10 are more susceptible to loss of signal when inappropriate buffers are used. With human T cells, the reduction in cytokine staining is milder with transcription factor buffer sets when performed optimally (Fig. 5B). For both mouse and human immune cells, we find that the dish soap protocol provides similar quality of cytokine staining as observed with kits optimized for intracellular cytokine staining while enabling detection of transcription factors. It should be noted, however, that stimulation with phorbol esters and ionomycin reduces the expression of some transcription factors and can also cause differential loss of some cell populations.

### Enhancement With Anti‐GFP

**Figure 6:**
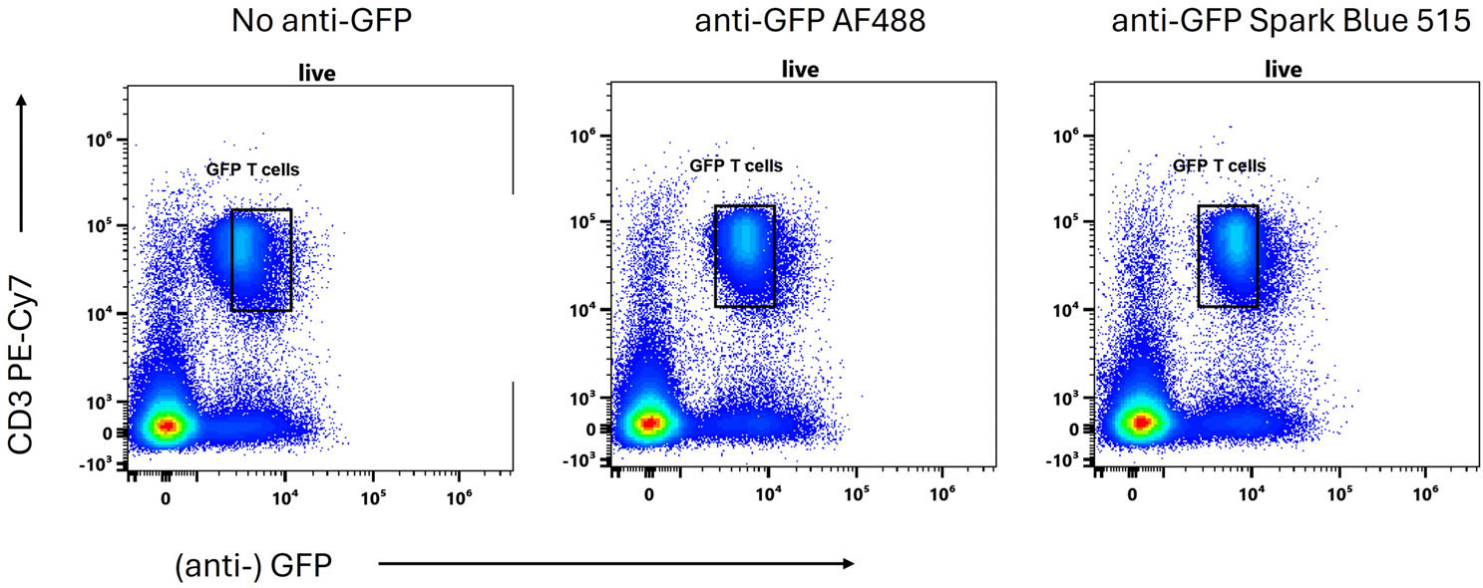
GFP signal amplification using anti‐GFP antibody. Comparison of GFP signal intensity with no amplification, anti‐GFP‐Alexa Fluor 488 amplification or anti‐GFP Spark Blue 515 amplification on mouse splenocytes processed with the dish soap protocol.

For weak fluorescent protein signals, amplification using antibody staining can enhance the signal‐to‐noise ratio. For this to work, the antibody must not increase the background noise, and we generally use the FM264G anti‐GFP clone (available from BioLegend) as we find it binds minimally to mouse and human cells in the absence of GFP. While the antibody can be conjugated to any number of fluorophores, a common choice in traditional flow cytometry is Alexa Fluor 488 due to its brightness and ability to add signal on top of the GFP in the same emission range. On many spectral cytometers, GFP and Alexa Fluor 488 are distinct enough to permit unmixing as separate channels. Using Alexa Fluor 488 on top of GFP is liable to create unmixing errors due to the difference in emissions. If the conjugated antibody's fluorescence is to be added to the fluorescent proteins in the same unmixed channel, care must be taken to match the spectral signatures carefully or allow for variation in the panel design. We have tested a Spark Blue 515 conjugated FM264G anti‐GFP and find it to be brighter than the corresponding Alexa Fluor 488 conjugate (Fig. 6) as well as being considerably closer to GFP's spectral signature (cosine similarity 0.96 for Spark Blue 515 and GFP as opposed to 0.92 for Alexa Fluor 488 and GFP on a 5‐laser Cytek Aurora).

### Note on Temperature

We have tested fixation at 4°, 21°, and 37°C (data not shown). At lower temperatures, nuclear staining is improved, but less GFP is retained. This may provide a better compromise for cells with brighter fluorescent protein signals. At 37°C, GFP preservation was optimal, but transcription factor staining was very poor. Thus, for general use, we recommend fixation at room temperature.

### Note on Phospho Staining

**Figure 7:**
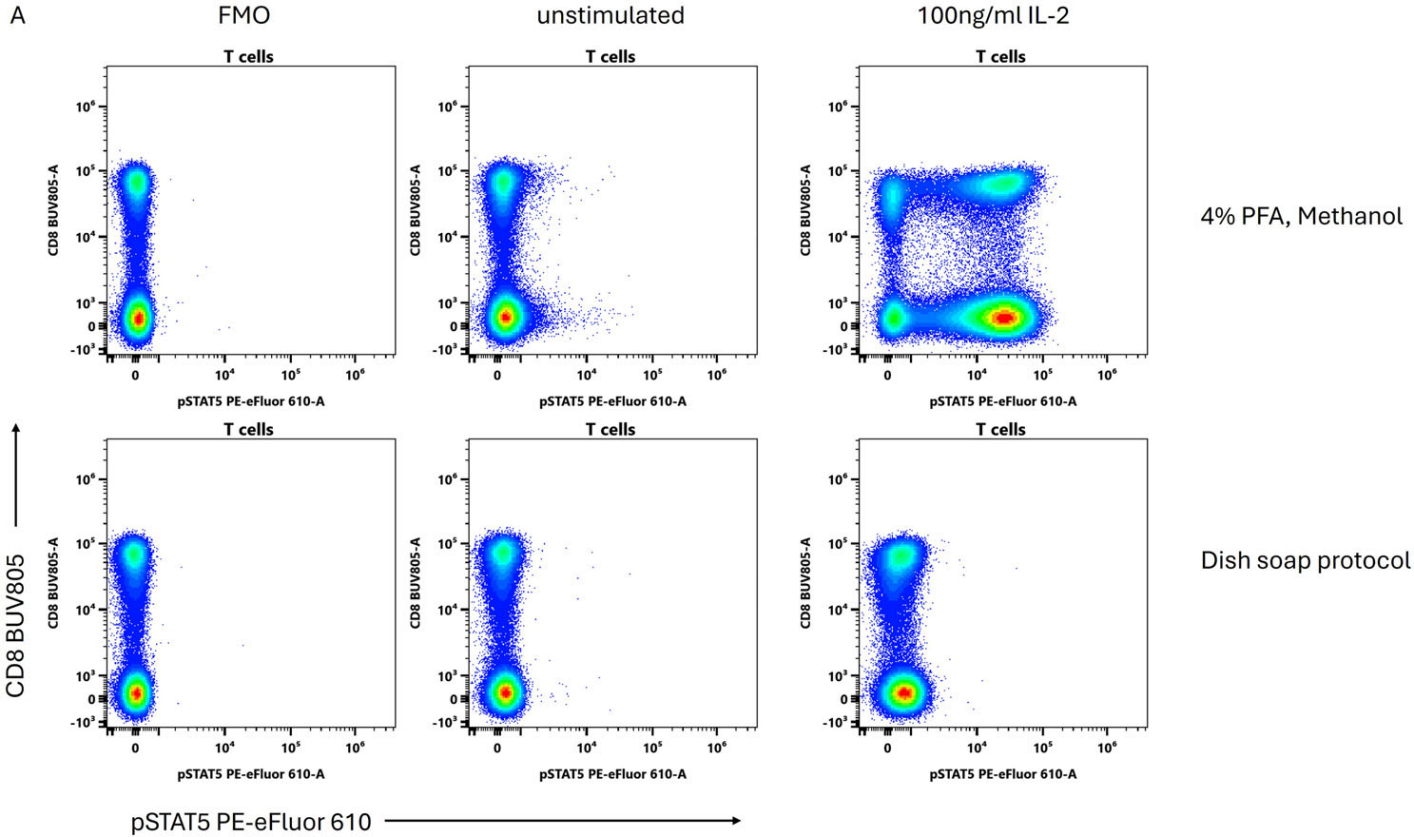
The dish soap protocol is not suitable for phospho‐flow. Human PBMC were stimulated with 100 ng/ml IL‐2 for 20 min prior to processing with the Krutzik protocol for phospho‐flow or the dish soap protocol. Samples were then stained overnight for pSTAT5. Plots are shown gated on viable single CD3^+^ T cells. Samples were stained for surface markers prior to stimulation.

Optimal staining for phosphorylated epitopes requires excellent permeabilization of the cell since many targets for phospho‐flow are transcription factors that translocate to the nucleus upon activation, e.g., the STAT family. Critically, however, staining of the phosphorylated state requires inactivation of endogenous phosphatases, and in the case of STATs, disruption of protein dimers. Methanol and other alcohols perform this function by dehydrating and denaturing cellular enzymes. While theoretically this function could be substituted by small molecule phosphatase inhibitors, we were unable to find a combination of cheap phosphatase inhibitors that worked anywhere nearly as effectively as methanol, and the dish soap protocol does not permit detection of phosphorylation states for STAT3, STAT5 or ERK (Fig. 7 and data not shown). Thus, we continue to recommend methanol for phospho‐flow. We note that the Krutzik protocol can be modified to achieve superior staining for phosphorylated epitopes and nuclear targets through the use of overnight staining (Krutzik & Nolan, 2003; Whyte et al., 2022).

### Troubleshooting

In Table 1, we provide a quick guide for the user to start on the path to identifying and reducing non‐specific interactions in their flow cytometry.

**Table 1:** Troubleshooting Guide for Unwanted Signals

Problem	Possible cause	Solution/steps
Minimal fluorescent protein signal after fixation	Inadequate fixation; fixation step skipped	Confirm that the formalin is buffered
		Test that signal remains after fixation without permeabilization
		Use fixative without Triton
		Increase fixation time (e.g., 45 min)
		Increase temperature of fixation (e.g., 25°C)
Minimal transcription factor staining	Inadequate staining	Use a brighter fluorophore (e.g., PE‐based dye or Real dye)
		Stain overnight
		Change panel design to improve resolution
Minimal transcription factor staining	Non‐specific binding	Check host species of transcription factor antibodies, block with sera from those species
		Block prior to staining
		Include FMO control(s)
Minimal transcription factor staining	No biological signal	Confirm expression in your cell type
		Assess both known positive and known negative cell populations (e.g., NK cell versus naïve T cell for T‐bet staining)

### Author Contributions

**Oliver Burton**: Conceptualization; investigation; methodology; writing—original draft. **James Dooley**: Conceptualization; methodology; project administration; supervision; writing—review and editing. **Adrian Liston**: Conceptualization; funding acquisition; project administration; resources; supervision; writing—review and editing.

### Conflict of Interest

Oliver Burton provides flow cytometry consulting services and has consulted for Bio‐Rad, makers of StarBright dyes. The other authors declare no conflict of interest.

## Data Availability

FCS files used to generate the figures have been deposited in Mendeley data at: Burton, Oliver; Dooley, James; Liston, Adrian (2025), “The Dish Soap Protocol”, Mendeley Data, V1, doi: 10.17632/khg36vdhnx.1 Burton, Oliver; Dooley, James; Liston, Adrian (2025), “The Dish Soap Protocol: Cytokines”, Mendeley Data, V1, doi: 10.17632/8t4sp6fnsd.1 Burton, Oliver; Dooley, James; Liston, Adrian (2025), “The Dish Soap Protocol: Phospho”, Mendeley Data, V1, doi: 10.17632/y8p5897n5f.1.
